# Editorial: Exploiting wheat biodiversity and agricultural practices for tackling the effects of climate change

**DOI:** 10.3389/fpls.2023.1257502

**Published:** 2023-08-18

**Authors:** Iris Aloisi, Ines Yacoubi, Agata Gadaleta, Andrés Ricardo Schwember, Ilaria Marcotuli

**Affiliations:** ^1^ Department of Biological, Geological, and Environmental Sciences, University of Bologna, Bologna, Italy; ^2^ Biotechnology and Plant Improvement Laboratory, Centre of Biotechnology of Sfax, Sfax, Tunisia; ^3^ Department of Soil, Plant and Food Science, University of Bari Aldo Moro, Bari, Italy; ^4^ Facultad de Agronomía e Ingeniería Forestal, Pontificia Universidad Católica de Chile, Santiago, Chile

**Keywords:** wheat biodiversity, agricultural practices, climate change, seed microbiome, wheat kernel weight, QTL

Wheat is a vital cereal crop globally, providing essential calories and protein to the human population, with the highest harvested area and the second-highest production rate. However, its quality depends on genetic variability, environmental conditions, cultivation practices, and their interactions, making it vulnerable to the impacts of climate change, such as rising temperatures and extreme weather events. In the dry regions of the Mediterranean area wheat is greatly vulnerable to several abiotic stresses such as hot temperatures, drought, and salinity, causing plant growth decrease together with severe yield and quality losses (Yacoubi et al., 2022). According to FAO projections, agricultural production requires to increase about 50% by 2,050 to meet the global rising demand for food (Food and Agriculture Organization of the United Nations), and in this context, the development of new durum wheat high-yielding cultivars and tolerant to abiotic stresses is highly necessary (Arriagada et al., 2022). To address this pressing issue, innovative approaches and cutting-edge technologies showcased in this Research Topic hold tremendous potential for securing food systems in the face of climate change. The identification of quantitative trait loci (QTL) associated with molecular markers is essential for understanding the genetic basis of important traits, and an effective method for improving selection efficiency in breeding programs (Colasuonno et al., 2016; Soriano et al., 2021). Hundreds of QTL, using both linkage analysis and genome-wide association studies (GWAS), have been mapped into the durum wheat genome, which have been summarized in previous works considering grain quality (Nigro et al., 2014; Marcotuli et al., 2020), and grain yield traits (Arriagada et al., 2020).These studies emphasize the urgency of the issue and demonstrate the power of agricultural innovation in transforming our food production systems to promote sustainable agriculture and ensure global food security. Aswini et al. conducted a study to investigate the potential beneficial functions of the seed microbiome in heat-tolerant and heat-susceptible wheat lines using high-throughput sequencing and culturable and unculturable approaches. The study found that the heat-susceptible variety had higher culturable diversity, with *Bacillus* being the dominant bacterial genus. In contrast, the heat-tolerant variety had thermophilic isolates and specific associations with thermophilic unculturable bacteria, which may contribute to enhanced resilience in plants. Metagenomic analysis revealed higher bacterial diversity in the seed microbiota, with *Proteobacteria* being the predominant phylum, followed by *Firmicutes* and *Actinobacteria*. The authors hypothesized that the heat-sensitive variety may have a higher population and diversity of culturable bacteria due to their preferential survival under heat-stressed conditions, which could explain the lower diversity of heat-tolerant varieties. They recommend exploring heat sensitivity variables in diverse agro-climatic zones to expand knowledge of microbial communities’ interaction with plants, their composition, and mechanisms that induce heat stress tolerance. The study underscores the potential of microbiome approaches to improve crop efficiency and support sustainable agriculture amid changing climatic conditions, highlighting the urgent need for innovative solutions in agriculture. Yannam et al. emphasized the importance of exploiting the inherent genetic diversity in bread wheat breeding programs. To this end, they investigated the use of wild relatives and landraces as a valuable approach in pre-breeding, leading to an enhancement of both quality and agronomic characteristics. In this study, the researchers conducted a genome-wide association analysis (GWAS) with PCA and kinship matrix, identifying 53 quantitative trait loci (QTL) hotspots containing 165 significant MTAs for 15 traits using 170 landraces from the Mediterranean basin. They also identified 807 CGs within these QTL hotspots, including ten specifically expressed in the grain, indicating their role in bread wheat quality and grain formation. A cross-validation approach was used to calculate the accuracies of genomic prediction for these traits, informing new breeding strategies. Genomic prediction accuracy for quality and agronomic traits ranged from -0.03 to 0.64 and 0.46 to 0.65, respectively. Prediction equations were developed to estimate bread loaf volume in landraces, with prediction abilities ranging from 0.67 to 0.82 depending on trait complexity. The study highlights the value of exploiting genetic diversity in landraces and wild relatives for pre-breeding in wheat breeding programs, enabling the development of precise and efficient breeding strategies through the identification of QTLs and candidate genes for enhanced quality and agronomic traits. Kumar et al. aimed to address the serious threat that climate change poses to food security by leveraging wheat genetic variability. The study evaluated 34 landraces and elite cultivars of *Triticum* spp. for phenological and yield-related traits under optimum, heat, and combined heat-drought stress environments. The researchers evaluated several phenological and yield-related traits, along with stress tolerance indices, to identify stress-tolerant and stress-susceptible lines. The study revealed a significant genotype x environment interaction, with the trait performance of genotypes exhibiting a significant reduction under combined heat-drought stress, resulting in the maximum seed yield penalty. Regression analysis highlighted the importance of the number of grains per spike in stress tolerance. Nonetheless, several genotypes were identified as stress-tolerant (heat and drought) in this study, indicating the potential of using selection indices and consistent association with physiological traits to increase the selection efficiency of superior genotypes. Overall, the findings of this study suggest that exploiting wheat genetic variability can be a promising approach to mitigate the impacts of climate change on food security. Identifying stress-tolerant genotypes can enable breeders to develop new varieties that can withstand the changing environmental conditions and secure food production. Wang et al. aimed to explore the responses of wheat kernel weight (KW) to diverse allelic combinations under projected climate warming conditions. The researchers focused on a subset of 81 wheat varieties with similar grain yield, biomass, and kernel number, and genotyped them at eight markers closely associated with thousand-kernel weight (TKW). They calibrated and evaluated the Agricultural Production Systems Simulator (APSIM-Wheat) model based on a unique dataset including phenotyping, genotyping, climate, soil physicochemistry, and on-farm management information. The researchers then used the calibrated model to estimate TKW under eight allelic combinations, seven sowing dates, and two climate scenarios (SSP2-4.5 and SSP5-8.5) driven by climate projections from five General Circulation Models (GCMs). The results showed that allelic combination, climate scenario, and sowing date significantly affected TKW. The interaction between allelic combination and climate scenario on TKW was also significant. The APSIM-Wheat model parameters aligned with the expression of the allelic combinations. Favorable allelic combinations mitigated the negative effects of climate change on TKW under projected scenarios. The study demonstrated that optimizing favorable allelic combinations can help achieve high wheat TKW and mitigate the negative effects of climate change on TKW. The findings of this study provide theoretical and practical reference for marker-assisted selection of high TKW in wheat breeding, which is important for improving wheat productivity under climate warming.

## Conclusion

The articles reported in this Research Topic highlight the transformative potential of agricultural innovation in addressing climate change challenges. Genetic diversity, digital technologies, and climate-smart crop management can build resilient, sustainable, high-quality food systems. However, realizing these innovations requires collaboration among scientists, farmers, policymakers, and stakeholders. Together, they can create a future where agriculture thrives amidst a changing climate, ensuring food security for all. Besides as reported in Colasuonno et al. (2021). To meet the food needs for the future, farmers must increase crop yields considering the climate and environment changes. Information gained from sequenced genomes in related species and in durum wheat, together with studies of fine mapping and QTL cloning, allows the identification of a high number of molecular markers, key genes, quantitative trait loci, and networks, which will lead to higher yielding crops.

## Author contributions

IA: Writing – original draft. IY: Supervision, Writing – review & editing. AG: Supervision, Writing – review & editing. AS: Supervision, Writing – review & editing. IM: Supervision, Writing – review & editing.
